# Supplementary material for: The Newly Identified Trichoderma harzianum Partitivirus (ThPV2) Does Not Diminish Spore Production and Biocontrol Activity of Its Host
Source: Viruses. 2022 Jul 14;14(7):1532. doi: 10.3390/v14071532 (PMC9317543; doi:10.3390/v14071532)
Supplement: Supplementary file 1 [file viruses-14-01532-s001.zip › viruses-1715247-supplementary.pdf]

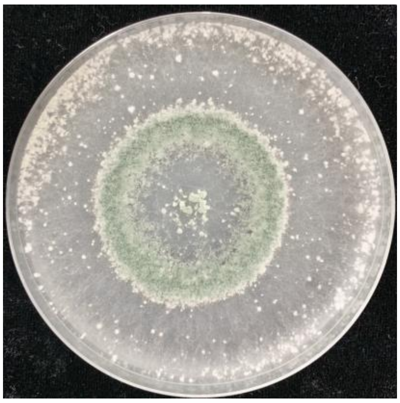

Figure S1. Colony morphology of *T. harzianum* strain T673 on PDA medium.

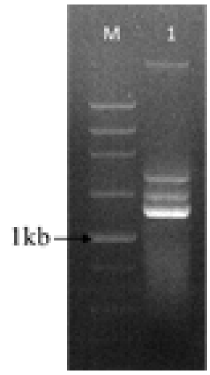

A.

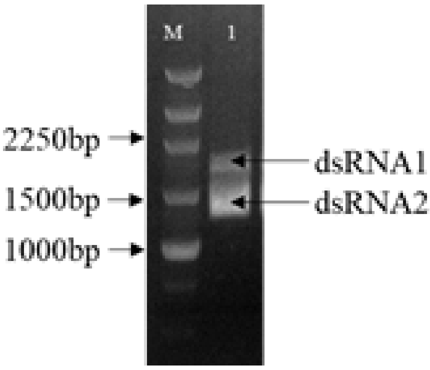

B.

Figure S2. The extraction of dsRNA from strain T673 and identification by enzyme digestion. A. DsRNA extracted from strain T673. M: DNA Marker (250 bp DNA Ladder); Line 1:DsRNAs detected from *T. harzianum* strain T673. B. DsRNA from T673 treated with by S1 nuclease and DNase IM: DNA Marker (250 bp DNA Ladder); Line 1: dsRNA digested by S1 nuclease and DNase I.

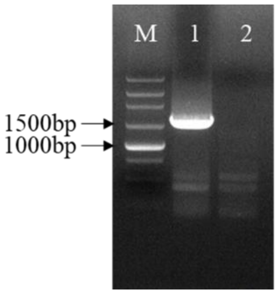

A.

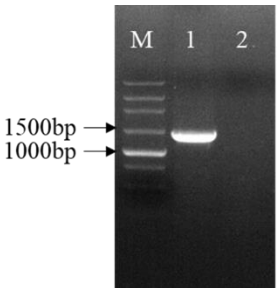

B.

Figure S3. Presence and absence ofThPV2 in strains T673 (before elimination) and T673-F (after elimination) respectively.A. Detection of ThPV2 dsRNA1 in strains T673 and T673-F by RT-PCR. M: DNA Marker (250 bp DNA Ladder); Line 1: Results from strain T673; Line 2: Results from strain T673-F; B. Detection of ThPV2- dsRNA2 in strains T673 and T673-F by RT-PCR. M: DNA Marker (250 bp DNA Ladder); Line 1: Results from strain T673; Line 2: RT-PCR from strain T673-F.

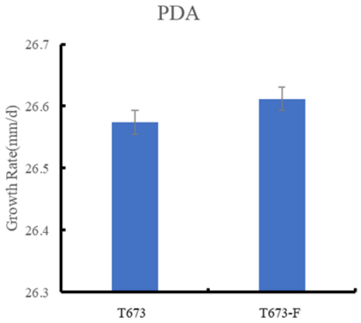

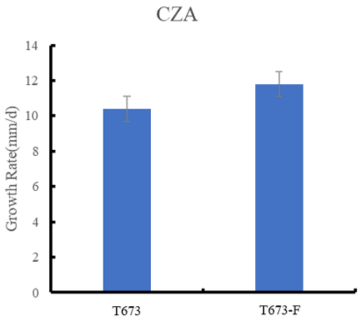

A B

Figure S4. Growth rates of strains T673 and T673-F on (A) PDA and (B) CZA mediums.

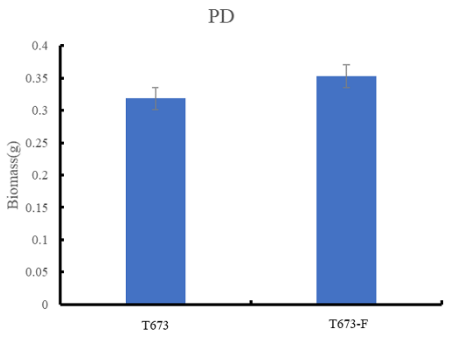

Figure S5. Biomass comparison of strains T673 and T673-F on PD medium.

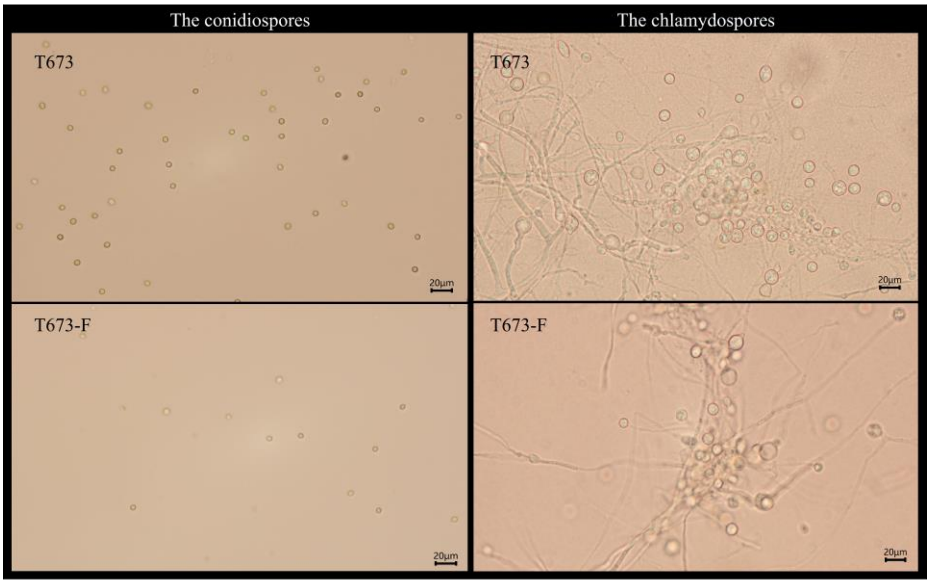

A **B**

Figure S6. Conidiospore and chlamydospore of T673 and T673-F. A: Conidiospore of T673 and T673-F produced in PDA medium; B: Chlamydospore of T673 and T673-F produced in chlamydospore induction medium.

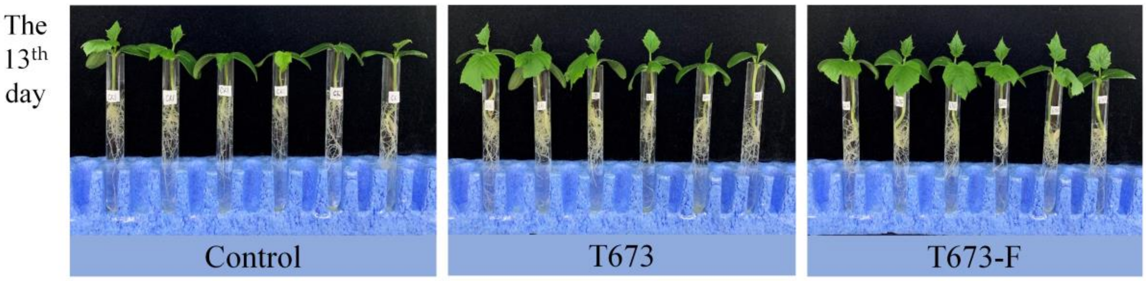

A

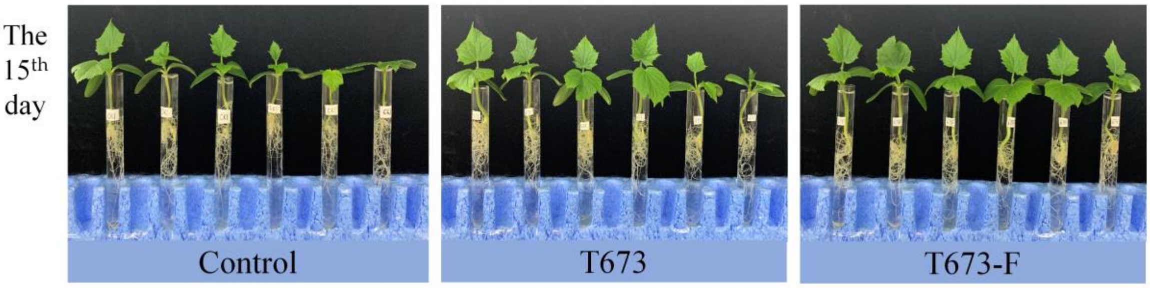

B

Figure S7. *T. harzianum* strain T673 and T673-F effects on plant growth. Growth of cucumber seedlings treated with spore suspension of T673, T673-F, or neither (control) on day 13 (A) and day 15 (B) post inoculation.

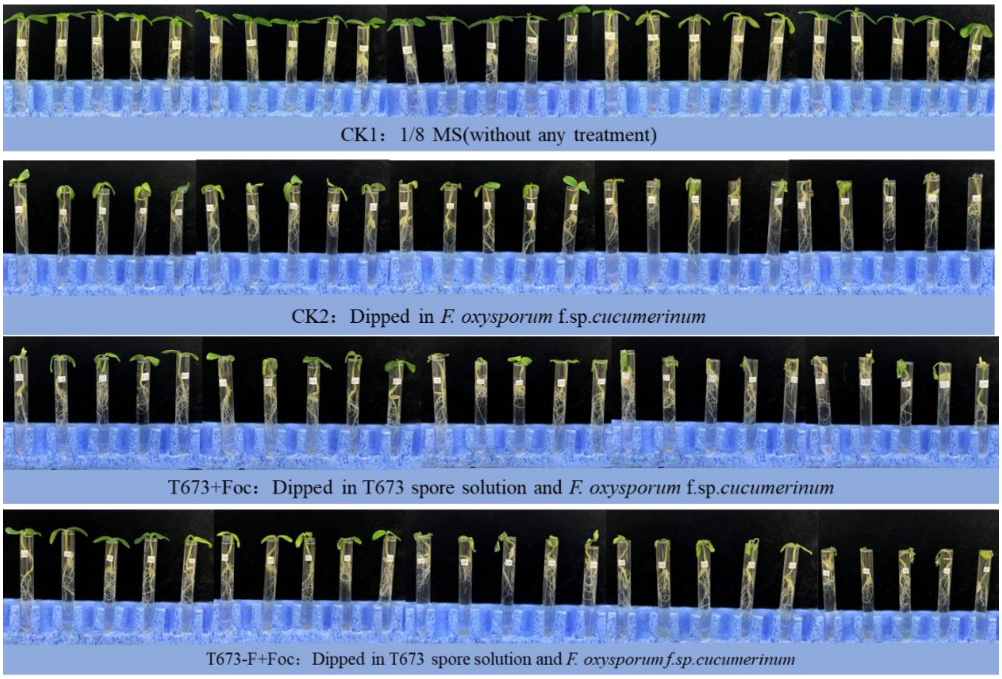

Figure S8. Biocontrol effects of strains T673 and T673-F against *F. oxysporum*f.sp.*cucumerinum* at 10 days after inoculation.

Table S1. The information of *Trichoderma* strains collected from soil of Xinjiang, Inner Mongolia, Jilin and Heilongjiang provinces of China

The order of the isolates	Strain Number	*Strain Name*	Region
112	CTCCSJ-G-QT40112	*Trichoderma.cf. harzianum*	Inner Mongolia
125	CTCCSJ-G-QT40125	*Trichoderma longibrachiatum*	Inner Mongolia
127	CTCCSJ-F-KY40127	*Trichoderma.cf. harzianum*	Inner Mongolia
130	CTCCSJ-G-QT40130	*Trichoderma.cf. harzianum*	Inner Mongolia

144	CTCCSJ-F-KY40144	*Trichoderma.cf. harzianum*	Inner Mongolia
154	CTCCSJ-G-QT40154	*Trichoderma longibrachiatum*	Inner Mongolia
155	CTCCSJ-F-KY40155	*Trichoderma longibrachiatum*	Inner Mongolia
169	CTCCSJ-F-ZY40169	*Trichoderma longibrachiatum*	Inner Mongolia
173	CTCCSJ-G-QT40173	*Trichoderma longibrachiatum*	Inner Mongolia
176	CTCCSJ-G-QT40176	*Trichoderma longibrachiatum*	Inner Mongolia
189	CTCCSJ-G-HB40189	*Trichoderma longibrachiatum*	Xinjiang
199	CTCCSJ-G-QT40199	*Trichoderma longibrachiatum*	Xinjiang
203	CTCCSJ-G-HB40203	*Trichoderma.cf. harzianum*	Inner Mongolia
206	CTCCSJ-G-HB40206	*Trichoderma.cf. harzianum*	Inner Mongolia
213	CTCCSJ-G-DK40213	*Trichoderma longibrachiatum*	Inner Mongolia
215	CTCCSJ-G-HB40215	*Trichoderma longibrachiatum*	Inner Mongolia
241	CTCCSJ-G-JK40241	*Trichoderma longibrachiatum*	Xinjiang
246	CTCCSJ-F-ZY40246	*Trichoderma afroharzianum*	Xinjiang
250	CTCCSJ-F-ZY40250	*Trichoderma afroharzianum*	Xinjiang
255	CTCCSJ-F-ZY40255	*Trichoderma longibrachiatum*	Inner Mongolia
264	CTCCSJ-G-QT40264	*Trichoderma longibrachiatum*	Inner Mongolia
272	CTCCSJ-G-QT40272	*Trichoderma longibrachiatum*	Inner Mongolia
273	CTCCSJ-G-HB40273	*Trichoderma longibrachiatum*	Inner Mongolia
277	CTCCSJ-G-HB40277	*Trichoderma longibrachiatum*	Inner Mongolia
291	CTCCSJ-F-ZY40291	*Trichoderma afroharzianum*	Xinjiang
300	CTCCSJ-F-ZY40300	*Trichoderma afroharzianum*	Inner Mongolia
303	CTCCSJ-G-QT40303	*Trichoderma asperelloides*	Xinjiang
320	CTCCSJ-G-QT40320	*Trichoderma hamatum*	Inner Mongolia
324	CTCCSJ-G-QT40324	*Trichoderma koningiopsis*	Inner Mongolia
330	CTCCSJ-G-QT40330	*Trichoderma gamsii*	Inner Mongolia
357	CTCCSJ-G-JK40357	*Trichoderma longibrachiatum*	Xinjiang
360	CTCCSJ-G-DK40360	*Trichoderma afroharzianum*	Inner Mongolia
366	CTCCSJ-G-QT40366	*Trichoderma longibrachiatum*	Inner Mongolia
368	CTCCSJ-F-KY40368	*Trichoderma longibrachiatum*	Inner Mongolia
388	CTCCSJ-G-QT40388	*Trichoderma afroharzianum*	Inner Mongolia
403	CTCCSJ-G-HB40403	*Trichoderma viridescens*	Xinjiang
405	CTCCSJ-G-HB40405	*Trichoderma saturnisporum*	Inner Mongolia
408	CTCCSJ-G-QT40408	*Trichoderma longibrachiatum*	Inner Mongolia
417	CTCCSJ-G-JK40417	*Hypocreasemiorbis*	Xinjiang
436	CTCCSJ-G-HB40436	*Trichoderma atroviride*	Xinjiang
441	CTCCSJ-G-HB40441	*Trichoderma harzianum*	Xinjiang
456	CTCCSJ-G-HB40456	*Trichoderma gamsii*	Xinjiang
457	CTCCSJ-G-HB40457	*Trichoderma guizhouense*	Inner Mongolia
459	CTCCSJ-G-HB40459	*Trichoderma.cf. harzianum*	Inner Mongolia
460	CTCCSJ-G-QT40460	*Trichoderma.cf. harzianum*	Inner Mongolia
465	CTCCSJ-G-QT40465	*Trichoderma.cf. harzianum*	Inner Mongolia

470	CTCCSJ-G-HB40470	*Trichoderma.cf. harzianum*	Inner Mongolia
477	CTCCSJ-G-HB40477	*Trichoderma.cf. harzianum*	Inner Mongolia
480	CTCCSJ-G-HB40480	*Trichoderma.cf. harzianum*	Inner Mongolia
483	CTCCSJ-G-HB40483	*Trichoderma harzianum*	Inner Mongolia
485	CTCCSJ-G-HB40485	*Trichoderma koningiopsis*	Inner Mongolia
491	CTCCSJ-G-HB40491	*Trichoderma.cf. harzianum*	Inner Mongolia
502	CTCCSJ-G-HB40502	*Trichoderma pleurotum*	Xinjiang
503	CTCCSJ-F-ZY40503	*Trichoderma citrinoviride*	Xinjiang
507	CTCCSJ-G-QT40507	*Hypocreapseudoharzianum*	Xinjiang
517	CTCCSJ-G-HB40517	*Trichoderma citrinoviride*	Xinjiang
519	CTCCSJ-G-HB40519	*Hypocreapseudoharzianum*	Xinjiang
536	CTCCSJ-G-HB40536	*Trichoderma longibrachiatum*	Xinjiang
537	CTCCSJ-G-HB40537	*Trichoderma harzianum*	Xinjiang
538	CTCCSJ-F-ZY40538	*Trichoderma citrinoviride*	Xinjiang
542	CTCCSJ-F-QT40542	*Trichoderma rossicum*	Xinjiang
550	CTCCSJ-G-HB40550	*Trichoderma asperellum*	Xinjiang
556	CTCCSJ-G-DK40556	*Trichoderma harzianum*	Xinjiang
558	CTCCSJ-G-DK40558	*Trichoderma harzianum*	Xinjiang
579	CTCCSJ-G-DK40579	*Trichoderma harzianum*	Xinjiang
623	CTCCSJ-G-QT40623	*Trichoderma harzianum*	Xinjiang
624	CTCCSJ-G-QT40624	*Trichoderma harzianum*	Xinjiang
639	CTCCSJ-F-KZ40639	*Trichoderma paraviridescens*	Xinjiang
642	CTCCSJ-F-KZ40642	*Trichoderma harzianum*	Xinjiang
646	CTCCSJ-G-JK40646	*Trichoderma harzianum*	Xinjiang
649	CTCCSJ-F-KZ40649	*Trichoderma harzianum*	Xinjiang
651	CTCCSJ-F-KZ40651	*Trichoderma harzianum*	Xinjiang
673	CTCCSJ-G-HB40673	*Trichoderma harzianum*	Xinjiang
703	CTCCSJ-F-KZ40703	*Trichoderma citrinoviride*	Xinjiang
713	CTCCSJ-G-HB40713	*Trichoderma rossicum*	Xinjiang
769	CTCCSJ-F-KZ40769	*Trichoderma paraviridescens*	Xinjiang
782	CTCCSJ-F-KZ40782	*Trichoderma harzianum*	Xinjiang
809	CTCCSJ-F-KZ40809	*Trichoderma harzianum*	Xinjiang
810	CTCCSJ-F-KZ40810	*Trichoderma harzianum*	Xinjiang
812	CTCCSJ-F-KZ40812	*Trichoderma paraviridescens*	Xinjiang
814	CTCCSJ-G-QT40814	*Trichoderma paraviridescens*	Xinjiang
816	CTCCSJ-F-KZ40816	*Trichoderma paraviridescens*	Xinjiang
827	CTCCSJ-F-KZ40827	*Trichoderma citrinoviride*	Xinjiang
835	CTCCSJ-G-QT40835	*Trichoderma asperellum*	Xinjiang
862	CTCCSJ-G-HB40862	*Trichoderma polysporum*	Xinjiang
878	CTCCSJ-G-HB40878	*Trichoderma harzianum*	Inner Mongolia
900	CTCCSJ-G-QT40900	*Trichoderma asperelloides*	Inner Mongolia
916	CTCCSJ-G-QT40916	*Trichoderma harzianum*	Inner Mongolia

920	CTCCSJ-G-HB40920	*Trichoderma harzianum*	Inner Mongolia
921	CTCCSJ-G-HB40921	*Trichoderma asperelloides*	Inner Mongolia
925	CTCCSJ-G-HB40925	*Trichoderma harzianum*	Inner Mongolia
927	CTCCSJ-G-HB40927	*Trichoderma harzianum*	Inner Mongolia
929	CTCCSJ-G-HB40929	*Trichoderma harzianum*	Inner Mongolia
930	CTCCSJ-G-HB40930	*Trichoderma harzianum*	Inner Mongolia
932	CTCCSJ-G-HB40932	*Trichoderma harzianum*	Inner Mongolia
934	CTCCSJ-G-HB40934	*Trichoderma harzianum*	Inner Mongolia
939	CTCCSJ-G-HB40939	*Trichoderma harzianum*	Inner Mongolia
940	CTCCSJ-G-HB40940	*Trichoderma asperelloides*	Inner Mongolia
941	CTCCSJ-G-HB40941	*Trichoderma harzianum*	Inner Mongolia
946	CTCCSJ-G-HB40946	*Trichoderma asperelloides*	Inner Mongolia
957	CTCCSJ-G-HB40957	*Trichoderma harzianum*	Inner Mongolia
961	CTCCSJ-F-KY40961	*Trichoderma harzianum*	Inner Mongolia
972	CTCCSJ-G-JK40972	*Trichoderma harzianum*	Inner Mongolia
974	CTCCSJ-G-JK40974	*Trichoderma harzianum*	Inner Mongolia
979	CTCCSJ-G-HB40979	*Trichoderma harzianum*	Inner Mongolia
980	CTCCSJ-G-JK40980	*Trichoderma harzianum*	Inner Mongolia
987	CTCCSJ-G-HB40987	*Trichoderma harzianum*	Inner Mongolia
988	CTCCSJ-G-HB40988	*Trichoderma longibrachiatum*	Inner Mongolia
989	CTCCSJ-G-HB40989	*Trichoderma harzianum*	Inner Mongolia
994	CTCCSJ-G-QT40994	*Trichoderma atroviride*	Heilongjiang
1012	CTCCSJ-G-DK41012	*Trichoderma harzianum*	Inner Mongolia
1023	CTCCSJ-G-HB41023	*Trichoderma harzianum*	Inner Mongolia
1038	CTCCSJ-G-HB41038	*Trichoderma harzianum*	Inner Mongolia
1039	CTCCSJ-G-HB41039	*Trichoderma harzianum*	Inner Mongolia
1044	CTCCSJ-G-HB41044	*Trichoderma harzianum*	Inner Mongolia
1045	CTCCSJ-G-HB41045	*Trichoderma harzianum*	Inner Mongolia
1047	CTCCSJ-F-KY41047	*Trichoderma harzianum*	Inner Mongolia
1050	CTCCSJ-G-QT41050	*Trichoderma rossicum*	Inner Mongolia
1053	CTCCSJ-G-HB41053	*Trichoderma harzianum*	Inner Mongolia
1060	CTCCSJ-G-JK41060	*Trichoderma harzianum*	Inner Mongolia

Table S2. The RPKM data of the virus species obtained from metagenomic sequencing.

unigene_ID	protein_description	E-value	673_RPKM
Contig7	YP_008327313.1 hypothetical protein N398_s2gp1 [Ustilaginoidea virens partitivirus 2]	8.30E-104	30374.55381
Contig7	AGO04404.1 hypothetical protein [Ustilaginoidea virens partitivirus]	1.20E-102	30374.55381
Contig7	AGJ03720.1 hypothetical protein [Ustilaginoidea virens mycovirus]	6.00E-78	30374.55381

Contig11	AGJ03719.1 RDRP [Ustilaginoidea virens partitivirus 3]	2.70E-202	36685.48928
Contig11	YP_008327312.1 RNA-dependent RNA polymerase [Ustilaginoidea virens partitivirus 2]	3.60E-199	36685.48928
Contig11	AGL42312.1 RNA-dependent RNA polymerase [Colletotrichum acutatum RNA virus 1]	1.80E-153	36685.48928
Contig26	YP_009272911.1 RNA-dependent RNA polymerase [Fusarium poae negative-stranded virus 1]	0.00E+00	4872.181635
Contig26	ALD89130.1 RNA-dependent RNA polymerase [Rhizoctonia solani negative-stranded virus 2]	1.10E-239	4872.181635
Contig26	ALD89111.1 RNA-dependent RNA polymerase [Rhizoctonia solani negative-stranded virus 3]	2.00E-230	4872.181635
Contig94	APG79235.1 RNA-dependent RNA polymerase [Beihai barnacle virus 5]	7.10E-151	768.8099433
Contig94	YP_009272912.1 RNA-dependent RNA polymerase [Fusarium poae negative-stranded virus 2]	6.20E-14	768.8099433
Contig7906	ACJ15076.1 nonstructural protein 1 [Influenza A virus (A/Muscovy duck/Vietnam/NCVD-22/2007(H5N1))]	2.50E-70	0.770317696
Contig7906	AAR99625.1 non-structural protein NS1 [Influenza A virus (A/duck/China/E319-2/03(H5N1))]	3.30E-70	0.770317696
Contig7906	ACB70828.1 nonstructural protein 1 [Influenza A virus (A/Muscovy duck/Vietnam/57/2007(H5N1))]	4.30E-70	0.770317696
Contig9788	ABN49709.1 protein H [Escherichia virus phiX174]	3.20E-48	0.657831378
Contig9788	ADM34807.1 protein H [Escherichia virus phiX174]	3.20E-48	0.657831378

Table S3. Primers used in this work

**Primer name**	**Primer sequence (from 5′ to 3′)**	**Used in the step of race**
Contig 7-F	TCAACATCCAAAACTACCGTTTTGTTAC	Whole contig seven
Contig7 -R	TCCAGGTTAACCCCTCATAGGTT	
Contig 11 -F	GACGCTCTTCCGATCTCTCA	Whole contig eleven
Contig 11 -R	TGTTCAAAAGTTCTCGACCCGTC	
C7-5′ primer A	TTCCCTCAAGTTCAAAGTGTCCGA	5′RACE (Reverse transcription)
C7-5′ primer B	ATGTTGCGGGCGGCTAAGAC	5′RACE (1st PCR)
C7-5′ primer C	TCAGCGGGAGCGGGGAC	5′RACE (2nd PCR)
C7-3′ primer A	GTCCGATTACCAACGCCTACTCTGT	3′RACE (1st PCR)
C7-3′ primerB	CTGCTCCCGGTCACGAACG	3′RACE (2nd PCR)
C11-5′ primer A	TGGCATACTCGATAAAGCATTGAAG	5′RACE (Reverse transcription)
C11-5′ primer B	CGGAACAACTTGGTCTGCACTGA	5′RACE (1st PCR)
C11-5′ primer C	ATTACTCGGCCTTGGCATTGC	5′RACE (2nd PCR)
C11-3′ primer A	CCTTCTTGTCGGATTTGGTCTATGAT	3′RACE (1st PCR)
C11-3′ primer B	CACGAGTGTAACCCTTGCAGCG	3′RACE (2nd PCR)

Table S4. The used RdRP and hypothetic protein data of mycovirues in the phylogentic analysis.

A. The used RdRP data of mycovirues in the phylogentic analysis.

Accession Number of RdRP	mycovirus name	The host	Similarity
QED88095.1	Colletotrichum gloeosporioidespartitiviru s 1	*Colletotrichum gloeosporioides*	76.4%
QHD64801.1	Plasmoparaviticola lesion associated Partitivirus 3	*Plasmoparaviticola*	72.8%
QJW70316.1	Erysiphe necator associated partitivirus 7	*Erysiphe necator*	72.6%
QHD64807.1	Plasmoparaviticola lesion associated Partitivirus 4	*Plasmoparaviticola*	71.6%
QKO02079.1	Macrophominaphaseolina partitivirus 1	*Macrophominaphaseolina*	69.1%
AGJ03719.1	Ustilaginoidea virens partitivirus 3	*Ustilaginoidea virens*	64.3%
YP_008327312.1	Ustilaginoidea virens partitivirus 2	*Ustilaginoidea virens*	62.7%
QDE53634.1	Aspergillus flavus partitivirus 1	*Aspergillus flavus*	48.5%
AGL42312.1	Colletotrichum acutatum RNA virus 1	*Colletotrichum acutatum*	48.9%
AIE47694.1	Botryosphaeria dothidea virus 1	*Botryosphaeria dothidea*	48.0%
QOL02536.1	Fusarium cerealispartitivirus 1	*Fusarium cerealis*	49.4%
QKK35392.1	Erysiphe necator associated gammapartitivirus 1	*Erysiphe necator*	31.7%
ABC86749.1	Aspergillus ochraceous virus	*Aspergillus ochraceous*	29.7%
AYP71818.1	Aspergillus ochraceous virus	*Aspergillus ochraceous*	30.1%
AXY93817.1	Medicago sativa alphapartitivirus 1	*Medicago sativa*	16.8%
AMW07365.2	Rhizoctonia solani dsRNA virus 3	*Rhizoctonia solani*	15.6%
BAV56297.1	Fusarium poae virus 1-240374	*Fusarium poae*	12.2%
YP_009177606.1	Rosellinianecatrixpartitivi rus 6	*Rosellinianecatrix*	13.0%
CBW77436.1	Fig cryptic virus	*Fig cryptic*	16.6%
AEJ07890.1	Pepper cryptic virus 1	*Pepper cryptic*	16.8%
ARS33771.1	Cryptosporidium parvum virus 1	*Cryptosporidium parvum*	17.2%

B. The used hypothetic protein data of mycovirues in the phylogentic analysis.

Accession Number ofhypothetical protein	mycovirus name	The host	Similarity
QED88096.1	Colletotrichum gloeosporioidespartitivirus 1	*Colletotrichum gloeosporioides*	63.6%
QJW70323.1	Erysiphe necator associated partitivirus 7	*Erysiphe necator*	60.5%
QDK65070.1	Phomamatteuciicolapartitivirus 1	*Phomamatteuciicola*	61.6%
QHD64811.1	Plasmoparaviticola lesion associated Partitivirus 4	*Plasmoparaviticola*	58.2%
YP_008327313.1	Ustilaginoidea virens partitivirus 2	*Ustilaginoidea virens*	54.6%
AGO04404.1	Ustilaginoidea virens partitivirus	*Ustilaginoidea virens*	54.6%
QKO02080.1	Macrophominaphaseolina partitivirus 1	*Macrophominaphaseolina*	52.3%
AGJ03720.1	Ustilaginoidea virens mycovirus	*Ustilaginoidea virens*	44.4%
AGL42313.1	Colletotrichum acutatum RNA virus 1	*Colletotrichum acutatum*	25.7%
QDE53635.1	Aspergillus flavus partitivirus 1	*Aspergillus flavus*	22.5%
AIE47695.1	Botryosphaeria dothidea virus 1	*Botryosphaeria dothidea*	23.9%
QOL02537.1	Fusarium cerealispartitivirus 1	*Fusarium cerealis*	20.5%
AXY93815.1	Medicago sativa alphapartitivirus 1	*Medicago sativa*	12.6%
AMW07366.2	Rhizoctonia solani dsRNA virus 3	*Rhizoctonia solani*	12.5%
BAV56298.1	Fusarium poae virus 1-240374	*Fusarium poae*	7.5%
CBW77437.1	Fig cryptic virus	*Fig cryptic*	12.0%
AEJ07891.1	Pepper cryptic virus 1	*Pepper cryptic*	11.6%
ARS33772.1	Cryptosporidium parvum virus 1	*Cryptosporidium parvum*	10.4%
BAT24480.1	Rosellinianecatrixpartitivirus 6	*Rosellinianecatrix*	6.7%

Table S5. The accession numbers for Trichoderma HarzianumPartitivirus 2 (ThPV2).

Accession number	The protein encoding
OL457022	Trichoderma HarzianumPartitivirus 2 (ThPV2)-RNA depended RNA polymerase
OL457023	Trichoderma HarzianumPartitivirus 2 (ThPV2)-hypothetical protein

Table S6 Growth rate of strain T673 and strain T673-F on PDA medium.

Repeats	Strain T673（mm/d）	Strain T673F（mm/d）
1	27.155	27.25
2	23.195	27.37
3	25.04	25.45
4	27.535	27.77
5	27.26	26.855
6	27.28	27.035
7	27.67	25.985
8	24.785	26.205
9	27.28	26.245
10	27.9	26.864
11	26.765	20.955
12	26.97	27
13	27.375	26.965
14	27.445	27.62
15	28.02	27.055
16	25.91	27
17	24.54	27.025
18	27.23	27.235
19	26.155	26.12
20	27.31	24.97
21	25.4	27.785
22	26.605	27.135
23	25.405	27.735
24	26.495	27.135
25	27.625	26.525
$\bar{x}$	26.574	26.61156
**Standard deviations**	1.223487161	1.369245993
**Statistical significance of strain T673 and T673-F**	0.9216127755＞0.05	

Table S7 Growth rate of strain T673 and strain T673-F on CZA medium.

Repeats	Strain T673 (mm/d)	Strain T673F (mm/d)
1	7.075	12.36
2	12.2375	10.575
3	11.1025	10.7675
4	10.63	11.075
5	13.245	11.8825
6	7.247	13.14
7	2.9375	10.8375
8	9.18	13.0425
9	12.0825	13.35
10	11.73	13.89
11	6.345	9.3825
12	9.0025	12.1425
13	7.91	12.58
14	7.9825	11.075
15	14.565	13.3
16	12.2375	11.54
17	15.8275	9.085
18	8.5725	14.025
19	13.7725	11.09

20	12.02	13.7175
21	9.6475	11.725
22	12.05	12.2075
23	9.825	8.965
24	10.7575	9.335
25	11.6475	14.005
$\bar{x}$	10.39238	11.8038
**Standard deviations**	2.868123033	1.5746946
**Statistical significance of strain T673 and T673-F**	0.038334154＜0.05	

Table S8 Biomass comparison, standard deviation and significant difference analysis of biomass between two strains strain T673 and T673-F.

Repeats	Strain T673（g）	Strain T673-F（g）
1	0.381	0.379
2	0.306	0.332
3	0.335	0.389
4	0.346	0.384
5	0.273	0.398
6	0.291	0.405
7	0.312	0.283
8	0.333	0.362
9	0.309	0.281
10	0.297	0.316
$\bar{x}$	0.3183	0.3529
**Standard deviations**	0.031130728	0.046710575
**Statistical significance of strain T673 and T673-F**	0.065502424＞0.05	

Table S9. Statistical analysis of conidiosporesporulation capacity of strains T673 and T673-F on PDA medium.

Repetitions	Medium number	Strain T673（$*10^8$per/ml）	Strain T673-F（$*10^8$per/ml）
Repetition 1	1	3	2.3
	2	3.55	0.85
	3	4.1	1.3
	4	6.35	1.65
	5	6.45	4.05
	6	4.9	1.75
	7	4.8	2.4
	8	4.8	2.4
Repetition 2	1	3.75	1.15
	2	3.1	1.25
	3	4.75	1.6
	4	4.7	1.5
	5	6.2	2.3
	6	4.3	2.55
	7	4.8	4.05
	8	5.3	2.1

Repetition 3	1	1.8	1.4
	2	4.05	1.15
	3	7.5	2.1
	4	5.55	1.7
	5	5.6	1.45
	6	6.3	2.4
	7	7.05	2.5
	8	3.4	2.75
**Statistical significance of strain T673 and T673-F（p）**		4.51796E-11＜0.01	

Table S10. Statistical analysis of chlamydospores sporulation of strain T673 and T673-F in chlamydospore induction mediumon the fifth day

Repetitions	Medium number	**Strain T673（*10^7per/ml）**	**Strain T673-F（*10^7per/ml）**
Repetition 1	1	7.25	2
	2	8.5	3
	3	6.25	2.5
	4	7.4	2.45
	5	6.1	2.9
	6	8.35	2.75
	7	7.15	2.1
	8	7.9	2.3
Repetition 2	1	7.55	2.15
	2	7.85	2.8
	3	6.9	2.35
	4	8.15	3.2
	5	7.7	2.4
	6	7.9	2.8
	7	5.95	2.2
	8	7.4	2.35
Repetition 3	1	7.1	2.75
	2	7.65	1.95
	3	7.6	2
	4	7.2	2.9
	5	7.35	2.65
	6	8	2.8
	7	7.25	2.7
	8	7.45	2.55
**Statistical significance of strain T673 and T673-F（p）**		1.68222E-33＜0.01	

Table S11. Statistical results of the growth promoting effects and reducing resistance of T673 and T673-F to *F. oxysporum*.f. sp. *cucumerinum* on cucumber planting in the glass tube.

A. Plain results of growth promoting effects of T673 and T673-F on cucumber planting in the glass tube on the 13th day (mm).

The number of days	Repetitions	Seedling number	Control			T673			T673F		
			The width of leave	The length of leave	The length of stem	The width of leave	The length of leave	The length of stem	The width of leave	The length of leave	The length of stem
The 13th day	Repetition 1	1	28.63	21.97	30.52	39.22	27.23	55.16	40.15	31.66	50.61
		2	21.38	19.53	30.25	32.49	22.07	36.07	37.24	26.39	66.25
		3	15.34	13.88	38.95	28.58	29.23	35.56	33.56	30.13	58.01
		4	14.95	11.85	27.12	18.61	16.36	35.84	32.47	26.19	56.25
		5	15.71	11.92	30.96	18.38	11.81	36.42	29.49	27.98	60.04
		6	12.80	14.16	33.04	23.60	17.56	40.68	22.93	20.98	55.20
		7	33.19	24.21	31.09	37.53	29.18	58.27	43.07	29.31	51.68
		8	26.85	22.79	42.12	36.63	26.77	37.91	37.32	26.08	49.59
	Repetition 2	1	23.295	22.51	33.82	34.96	28.96	39.04	40.56	29.98	52.87
		2	29.167	23.717	34.24	35.7	25.74	57.94	35.09	31.37	58.07
		3	26.491	22.33	33.18	34.67	30.44	38.17	33.13	34.43	51.80
		4	17.31	14.82	33.54	31.22	24.94	36.67	34.17	20.28	46.65
		5	16.7	12.46	29.95	31.36	30.87	42.53	34.09	26.95	51.01
		6	13.09	15.25	39.15	26.44	20.86	36.46	33.55	28.84	42.87
		7	16.3	18.01	32.55	23.37	19.18	41.18	32.02	30.57	46.61
		8	20.93	18.69	40.12	29.73	23.19	37.63	33.03	28.25	38.20
	Repetition 3	1	10.85	9.58	26.81	26.21	25.70	32.58	29.97	27.25	40.56
		2	15.05	12.79	23.94	23.85	24.63	37.27	30.31	30.01	38.42
		3	15.74	14.86	37.74	20.47	17.70	35.97	24.98	20.53	43.42
		4	13.33	14.58	24.89	19.36	14.96	34.80	24.79	23.02	39.03
		5	11.53	13.43	35.38	16.99	18.67	36.04	25.49	24.97	47.37

		6	14.73	14.04	30.64	16.98	17.20	34.62	36.95	29.65	39.62
		7	17.02	13.11	27.12	18.69	13.04	47.67	22.16	21.81	33.04
		8	18.21	17.59	25.53	14.24	14.97	29.55	23.06	23.85	40.09

B. Plain results of growth promoting effects of T673 and T673-F on cucumber planting in the glass tube on the 15th day (mm).

The number of days	Repetitions	Seedling number	Control			T673			T673F		
			The width of leave	The length of leave	The length of stem	The width of leave	The length of leave	The length of stem	The width of leave	The length of leave	The length of stem
The 15th day	Repetition 1	1	34.84	24.44	31.78	42.15	30.71	56.64	41.28	32.84	51.85
		2	30.38	22.60	30.43	37.44	26.48	37.12	40.36	34.79	66.55
		3	20.39	17.21	39.56	34.99	33.31	37.41	39.12	37.52	58.88
		4	17.76	16.49	27.29	23.58	24.19	38.03	38.05	32.70	57.10
		5	19.10	16.94	32.58	21.59	16.22	38.73	35.98	33.54	61.99
		6	21.93	18.11	33.14	28.36	25.73	40.78	27.69	26.26	56.57
		7	39.78	30.29	33.3	41.48	35.55	59.03	44.04	30.25	51,99
		8	34.08	27.03	43.28	41.64	36.05	40.27	39.49	32.97	50.13
	Repetition 2	1	29.86	27.93	35.84	37.51	33.2	40.04	45.83	33.77	52.94
		2	34.17	29.22	35.73	39.69	32.17	59.31	37.6	32.85	59.62
		3	30.91	26.60	33.62	39.09	34.63	39.11	37.33	35.28	52.97
		4	21.47	17.28	36.07	34.82	30.99	36.90	35.19	27.76	47.18
		5	23.38	18.36	30.85	35.24	31.67	42.96	38.01	32.64	52.71
		6	20.03	19.40	39.36	31.04	28.4	38.21	38.52	33.49	43.51

		7	19.69	22.45	32.98	28.88	26.75	43.26	36.27	35.05	48.69
		8	23.72	21.51	40.24	32.12	25.16	37.83	37.48	34.59	38.43
	Repetition 3	1	11.36	10.49	27.18	33.54	30.53	33.62	34.52	31.06	41.75
		2	19.79	16.34	25.01	27.73	25.18	37.81	32.73	31.61	39.2
		3	20.65	20.99	38.58	27.64	26.52	36.17	29.47	25.63	45.03
		4	14.21	16.22	24.99	26.77	20.19	35.19	30.85	28.63	40.2
		5	12.90	16.7	35.36	23.66	24.71	36.79	30.45	28.62	47.53
		6	16.51	18.46	32.13	21.47	20.38	35.06	38.27	34.09	40.86
		7	23.82	17.59	30.90	23.94	19.29	47.81	27.61	25.1	36.62
		8	22.19	20.33	26.04	15.35	15.01	30.6	28.47	27.08	40.33

C.Plain results of growth promoting effects of T673 and T673-F on cucumber planting in the glass tube on the 35th day (mm).

The number of days	Repetitions	Seedling number	Control			T673			T673F		
			The width of leave	The length of leave	The length of stem	The width of leave	The length of leave	The length of stem	The width of leave	The length of leave	The length of stem
The 35th day	Repetition 1	1	43.26	36.73	34.43	43.72	33.94	56.8	42.56	37.01	52.35
		2	37.06	39.94	30.85	40.91	32.73	37.26	41.97	41.26	66.68
		3	39.29	34.11	39.19	39.12	38.42	40.44	40.09	39.47	61.8
		4	28.75	33.35	29.57	36.05	31.54	39.1	40.69	39.17	58.95
		5	25.3	24.28	37.66	34.060	34.71	41.63	41.820	45.56	62.01
		6	25.47	25.27	34.70	39.540	38.72	42.9	38.59	35.95	56.71
		7	43.64	38.53	34.83	42.59	39.60	59.21	46.32	44.51	52.88
		8	39.72	38.57	44.97	43.68	36.92	40.97	41.68	39.07	52.19

	Repetition 2	1	32.54	30.68	36.02	39.69	33.47	40.35	48.37	47.07	53.23
		2	41.59	39.07	35.98	41.94	33.60	59.42	40.59	39.81	60.85
		3	36.28	34.88	33.81	39.86	35.17	40.01	42.57	40.18	52.99
		4	34.95	31.27	37.01	36.92	32.44	37.29	38.61	37.11	48.67
		5	34.55	33.16	30.63	42.15	35.67	43.83	42.58	30.64	53.18
		6	32.91	30.52	40.41	33.96	31.04	38.36	40.88	38.67	45.97
		7	27.86	26.30	33.60	35.03	31.26	43.67	40.15	40.01	50.77
		8	26.81	23.79	41.41	38.64	31.22	38.12	42.96	40.61	39.21
	Repetition 3	1	20.37	18.36	27.09	42.2	33.92	33.71	43.75	42.88	42.46
		2	27.04	26.51	25.73	35.59	30.64	38.03	37.64	37.89	40.79
		3	27.90	25.16	38.63	34.62	32.51	36.47	34.79	32.61	47.58
		4	23.46	20.17	25.67	32.85	29.66	35.3	38.45	35.60	42.01
		5	19.75	19.44	35.89	27.19	25.41	36.82	41.94	37.71	49.72
		6	23.12	22.43	33.70	25.90	23.47	35.34	43.81	42.22	41.98
		7	27.06	25.18	30.61	29.88	26.19	47.93	38.84	36.41	37.08
		8	28.72	26.11	27.77	20.07	16.07	30.87	37.1	32.05	41.43

D. Significant difference about the growth promoting effects of T673 and T673-F on cucumber planting in the glass tube on the 13th, 15th and 35th day (mm).

	On the 13th day			On the 15th day			On the 35th day		
	Control/673	Control/673F	T673/T673F	Control/673	Control/673F	T673/T673F	Control/673	Control/673F	T673/T673F
p (The width of leave)	0.0002509	7.74462E-10	0.0080878	0.0007087	1.37213E-08	0.0115461	0.0071858	9.01751E-08	0.0016135
p (The length of leave)	0.0005496	1.58416E-11	0.0011331	0.0001096	8.8503E-12	0.0030422	0.1250579	2.41907E-07	5.89155E-06

(The length of stem)	0.0001711	2.2357E-10	0.0006000	0.0001608	3.50508E-10	0.0008010	0.0002873	7.39557E-11	0.0002083

F. Situation of cucumber flowering and bud of T673 and T673-F in the glass tube on the 25th and 35th day.

Repetitions	Treatment	Index	The number of flowering on 25th day	The number of floweringon 35th day
Repetition 1	Control	The number of flowering	0	0
		The number of bud	0	1
		The number of non-flowering	8	7
	T673	The number of flowering	0	2
		The number of bud	3	3
		The number of non-flowering	5	3
	T673F	The number of flowering	0	1
		The number of bud	2	5
		The number of non-flowering	6	2
Repetition 2	Control	The number of flowering	0	0
		The number of bud	0	0
		The number of non-flowering	8	8
	T673	The number of flowering	0	4
		The number of bud	5	3
		The number of non-flowering	3	1
	T673F	The number of flowering	0	0
		The number of bud	2	3
		The number of non-flowering	6	5
Repetition 3	Control	The number of flowering	0	0

		The number of bud	0	0
		The number of non-flowering	8	8
	T673	The number of flowering	0	3
		The number of bud	4	2
		The number of non-flowering	4	3
	T673F	The number of flowering	0	0
		The number of bud	2	5
		The number of non-flowering	6	3

G. The difference statistics analysis of control/T673/T673-F about flowering and buds.

Condition	Control/T673	Control/T673-F	T673/T673F
The number of flowering and buds	0.0020187	0.0100597	0.4168655
The number of non-flowering and buds	0.0020187	0.0100597	0.4168655

TTable S12. Statistical results of antagonistic experiment of strains T673 and T673-F against six pathogens.

Pathogens	Control group pathogen colony radius（mm）	Colony radius of pathogenic after T673 treatment（mm）	Inhibition rate of strain T673（%）	Colony radius of pathogenic after T673-F treatment（mm）	Inhibition rate of strain T673-F（%）
Alternaria solanacearum	27.30	9.17	67.54	10.25	62.57
		8.90		10.07	
	30.55	10.04		10.95	
		9.45		12.04	
*Fusarium. oxysporum*f.sp.*cucumerinum*	39.38	11.42	69.19	12.85	67.60
		13.38		12.93	
	39.85	12.16		13.62	
		11.86		11.94	
Botrytis cinerea	66.67	19.97	74.44	18.41	72.34
		18.85		17.88	
	69.05	14.64		19.75	
		15.91		19.10	
Fusariumpseudograminearum	60.72	16.64	71.14	18.36	70.28
		18.21		18.75	
	58.54	16.62		16.66	
		17.37		17.12	
Phytophthora capsici	41.91	11.41	70.67	13.02	69.07
		11.38		12.78	
	41.52	13.47		12.87	
		12.68		12.94	
Rhizoctoniasolani	31.27	7.46	70.77	7.35	70.91
		7.97		8.66	
	30.25	11.07		9.04	
		9.47		10.75	

Table S13. Situation of cucumber planted in the glass tube for resistance to *F. oxysporum* f.sp.*cucumerinum* (FOC) treated by control, FOC, T673+FOC and T673-F+FOC respectively on the 13th day.

A. Disease levels of cucumber root rat disease infected by *FOC* on the 13th day

Disease level	Symptom description
Level 0	No symptoms
Level 1	The leaves do not wither and turn slightly yellow
Level 2	The leaves wither and the roots shrink slightly
Level 3	All the leaves withered, the roots turned brown and shrank
Level 4	The plant withered and died

B. Disease levels of cucumber infected by *FOC* on the 13th day

The 13th day															
	Repetition 1					Repetition 2					Repetition 3				
Disease level	Level 0	Level 1	Level 2	Level 3	Level 4	Level 0	Level 1	Level 2	Level 3	Level 4	Level 0	Level 1	Level 2	Level 3	Level 4
CK1	8					8					8				
FOC					8					8					8
FOC+T673			3		5			3		5			2		6
FOC+T673-F			2		6			1		7			1		7

C. Disease index of the treatments of cucumber infected by *FOC* on the 13th day

Treatment	Disease index on the 13th day			
	Repetition 1	Repetition 2	Repetition 3	Everage
FOC	100	100	100	100
FOC+T673	81.25	81.25	87.5	83.33
FOC+T673-F	87.5	93.75	93.75	91.67
